# Endoscopic ultrasound-guided injection-assisted precise localization for endoscopic removal of a penetrating foreign body in the gastric antrum: a case report

**DOI:** 10.1055/a-2808-7503

**Published:** 2026-03-05

**Authors:** Tian-Xing Yuan, Long Huang, Miao-Miao Li, Yu Bao, Rui Zhao

**Affiliations:** 1 University of Electronic Science and Technology of China, School of Medicine, Chengdu, China; 2 Department of Spleen and Gastroenterology, Chinese Medicine Hospital of Zhong Jiang, Deyang, China; 3 92293 Department of Endoscopy, Sichuan Cancer Hospital and Research Institute, Chengdu, China

A 61-year-old woman presented with a 2-week history of a foreign body at the lesser curvature of the gastric antrum after accidental fish bone ingestion, accompanied by upper abdominal pain radiating to the back.

**Fig. 1:**
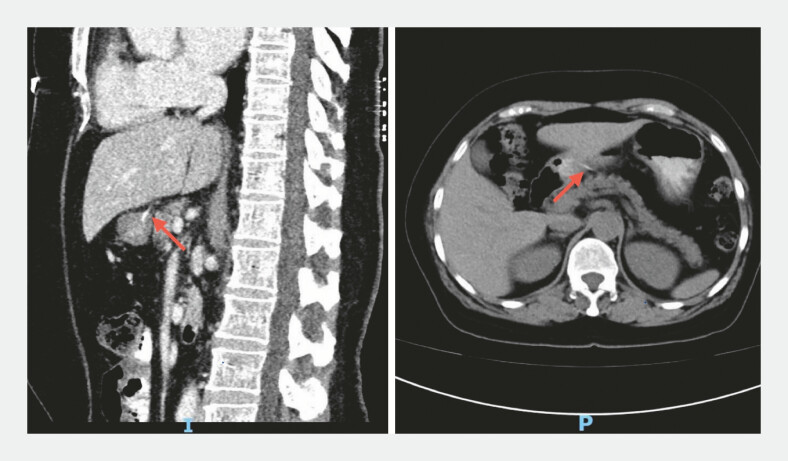
Contrast-enhanced computed tomography showed high-density linear shadows of the antrum and penetrated the stomach wall into the abdominal cavity.

**Fig. 2:**
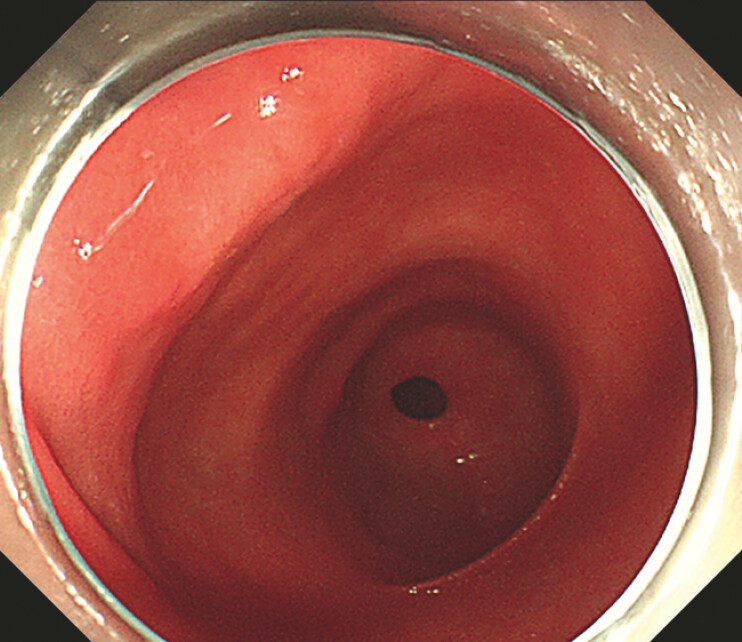
No significant abnormalities were observed under endoscopy.

**Fig. 3:**
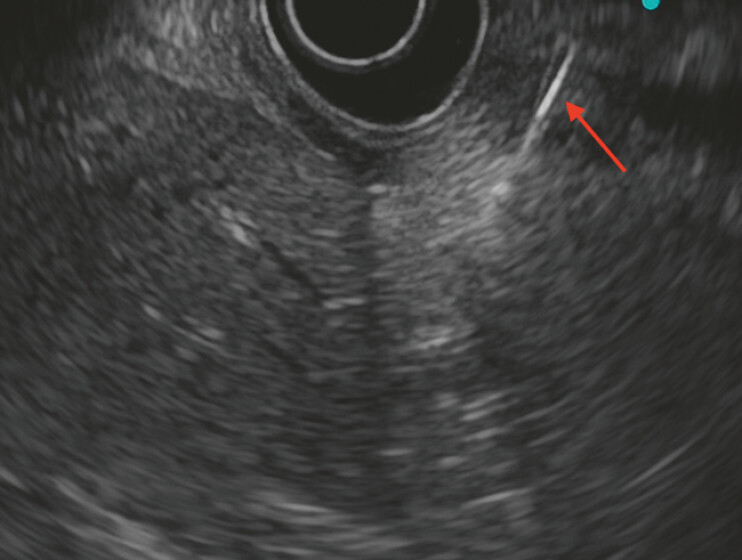
Endoscopic ultrasound shows the location and depth of the fishbone.

Contrast-enhanced computed tomography revealed a linear hyperdense foreign body in the gastric antrum penetrating the gastric wall into the peritoneal cavity ( Fig. 1 ). Conventional gastroscopy showed no obvious abnormalities ( Fig. 2 ). Endoscopic ultrasound demonstrated a linear foreign body embedded within the wall of the lesser curvature of the gastric antrum ( Fig. 3 ).

**Fig. 4:**
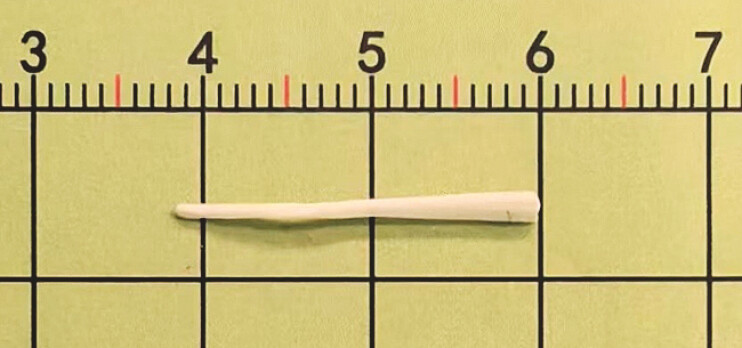
A 2.2cm fish bone.

Based on the clinical history and imaging findings, endoscopic submucosal dissection (ESD) was planned. Under endoscopic ultrasound (EUS) guidance, methylene blue was injected to localize the lesion. A focal mucosal incision at the marked site revealed a whitish fibrotic capsule, within which the distal end of the foreign body was exposed and gently extracted with forceps. A white, triangular-shaped foreign body was removed ( Fig. 4 ). No residual foreign material was observed, and the defect was closed with titanium clips. ( Video 1 ).

Injection under the guidance of endoscopic ultrasound assists the endoscope to remove the penetrating foreign body of the antrum.Video 1

Fish bones penetrating the gastric wall are often insidious and may not produce obvious mucosal changes on conventional endoscopy, leading many cases to be managed surgically or via laparoscopy 1 . Previous studies have also highlighted the critical role of EUS in locating deeply embedded or impacted foreign bodies, assessing penetration depth, and guiding subsequent interventions 2 . However, most reports describe the need for full-thickness ESD or surgical exposure after localization. In contrast, in our case, EUS-guided methylene blue injection enabled precise surface localization, allowing successful removal through a limited mucosal incision without extensive submucosal dissection or surgical intervention. This approach is both feasible and simplified, suggesting that EUS-assisted targeted localization may substantially reduce procedural complexity and offer a minimally invasive alternative for managing deeply embedded gastric foreign bodies.

Endoscopy_UCTN_Code_TTT_1AO_2AL
